# Relationship between radioactivity and toxicity in some medicinal plants

**DOI:** 10.1038/s41598-023-37403-7

**Published:** 2023-07-06

**Authors:** Eman H. Abdelfadeel, E. S. Abd El-Halim, T. M. Hegazy, H. A. Abdel Ghany

**Affiliations:** Department of Physics, Faculty of Women for Arts, Science and Education, Ain-Shams University, Cairo, Egypt

**Keywords:** Environmental sciences, Natural hazards

## Abstract

Plants absorb water, nutrients and minerals from the soil through their root. Also, minerals, the radionuclides present in the growing media also are absorbed by plant parts following the same pathway. Consequently, it is important to determine the concentrations of these radionuclides in edible plants to access the associated risk to human health. In the present work, the levels of natural radioactivity and the level of some toxic elements in 17 medicinal plants, commonly used in Egypt, were measured using high-purity germanium gamma spectrometry and atomic absorption, respectively. The investigated plants were sub-grouped according to the edible parts into leave samples (n = 8), roots (n = 3), and seeds (n = 6). Also, the specific activity of both radon and thoron was measured by using alpha emitters registration which is emitted from radon and thoron gases in CR-39 nuclear track detectors. Additionally, the concentration of some toxic elements (Cu, Zn, Cd and, Pb) in six samples of medicinal plants was determined by atomic absorption spectrometry.

## Introduction

There are naturally occurring radioactive materials (NORM) in all elements of the environment, including air, water, soil, food, and people. The NORMs in the environment mostly come from the decay chains of Uranium (^238^U) and Thorium (^232^Th)^1,2^. Radionuclides present in soil are usually transferred to different types of edible plant tissues and so to the human food chain. This phenomenon is called soil–plant transfer factor (TF) which is regarded as one of the most important parameters used in environmental safety assessment to estimate the amount of radioactivity that could be present in agricultural crops and estimate dose impact on the human health^3^. The transfer factor of radionuclides such as uranium, thorium, radium, and radon from soil to plants and their distribution among different parts of the plant depends on the soil nature and fertilizers. The latter are considered as technologically enhanced natural radiation, which increase the environmental uranium and partially thorium concentrations in the agricultural environment^4^. Medicinal plants are increasingly used in the developing countries because they are readily available and cheaper than modern medicines^5,6^. Other studies demonstrated that alternative medicine users consume medicinal plants because they are more congruent with their own values, beliefs, and philosophical orientations toward health and life, regardless their economical status^5^. Some studies suggested that the uptake of radionuclides in the plant depends on the type of plant^7^. Many medicinal plants are directly and indirectly used in the synthesis of medicines and supplements^8^. Accordingly, the content of radionuclides in medicinal plants contributes to the increase of the internal effective dose that predispose to the increased risks of developing lung cancer by emitting alpha particles^9^. Radon gas, with a half-life time of 3.8 days, is generated from the radioactive decay of radium -226 in the uranium transformation decay in the earth’s crust^10^. So, long term use of these plants may cause health hazards for the population and may be associated with most forms of blood cancers (leukemia) and cancers of some other organs such as the bone, lung, breast, and, thyroid^11^. Thus, estimation of radioactivity in different medicinal plants is of great importance and interest in health physics^12^. So, the aim of this work is to assess the natural radioactivity (^238^U, ^226^Ra, ^232^Th, ^40^K, ^222^Rn, and ^220^Rn) and its radiological hazards in some medicinal plants commonly used and to investigate the relationship between radionuclides and toxic elements in some of the samples studied.

## Materials and methods

### Ethics statement

The plant collection and use was in accordance with all the relevant guidelines**.**

### Sampling

Seventeen samples of medicinal herbal plants, commercially available in the local market were collected from. These samples are commercially available as powdered or smashed herbs, where they were coded from P1 to P17 for the study purpose. The investigated plants were selected due to their prevailing use for either protective or curative purposes in a non-conventional medical manner, particularly after the emergence of the Covid-19 pandemic. Medicinal plants under investigation were divided into three categories including seeds (P1, P5, P7, P11, P14 and, P16), roots (P3, P12, and P17), and leaves (P2, P4, P6, P8, P9, P10, P13, and P15) as shown in Table 1. Specialized member of the department of botany assigned the scientific name (Also known as binomial name) of these plants. The investigated samples were sieved through − 200 mesh size. Weighted samples were placed in polyethylene bottles of 250 cm^3^ volume. To prevent the possible escape of radon, the bottles were sealed tightly and stored for 4 weeks to reach secular equilibrium between ^226^Ra and ^232^Th with their progenies^13–15^.

**Table 1:** Description of the investigated plant samples.

Sample code	Traditional name	Scientific name	Part used	Pictures
P1	Cinnamon	*Cinnamomum verum*	Seeds	
P2	Thyme	*Thymus vulgaris*	Leaves	
P3	Ginger	*Zingiber officinale*	Roots	
P4	Sage	*Salvia officinalis*	Leaves	
P5	Camel thistle	*Silybum marianum*	Seeds	
P6	hyssop	*Hyssopus*	Leaves	
P7	Anise	*Pimpinella anisum*	Seeds	
P8	Chamomile	*Matricaria chamomilla*	Leaves	
P9	Telugu	*Tilia europea*	Leaves	
P10	Marjoram	*Origanum majorano*	Leaves	
P11	Carnation	*Syzygium aromaticum*	Seeds	
P12	Turmeric	*Curcuma longa*	Roots	
P13	Fennel	*Foeniculum vulgare*	Leaves	
P14	Black cumin	*Nigella sativa*	Seeds	
P15	Mint	*Menthe*	Leaves	
P16	Cestus	*Saussure cestus*	Seeds	
P17	Alpinia	*Alpinia officinarum*	Roots	

### Measurements

The natural radioactivity content of selected samples associated with ^238^U, ^226^Ra, ^232^Th, and ^40^K was analyzed using a high purity germanium detector. The detector was connected with electronic circuits which consist of a preamplifier, amplifier, and power supply. The detector has a resolution (FWHM) of 1.85 for the ^60^Co 1332.2 keV gamma-ray line. The efficiency calibration for the HPGe detector was carried out using a ^226^Ra point source. The relative efficiency curve was normalized for the 250 ml capacity beakers by the concentration of chemically pure potassium chloride solution in distilled water. Similar sample size was used to obtain the absolute efficiency curve. The detector was surrounded with lead shielding to reduce the interference with background radiation. The activity concentrations were calculated as follows:

- ^238^U concentrations were determined by measuring the 295.1 (19.2%), 352 (37.2%) keV γ- rays from ^214^Pb, the 609.3 (46.1%) and 1120.3(15.1%) keV γ-rays from ^214^Bi.
- ^232^Th activity was determined from the γ-peaks of 238.6 (43.6%) keV from ^212^Pb, 911.2 (29.0%) and 969.0 (23.2%) keV from ^228^Ac, and 583.0 (31.0%) keV γ-rays from ^208^Tl.
- ^40^K concentration was measured from its 1460 (10.7%) keV γ-line.
- ^226^Ra concentration was determined by measuring the γ peak of 186 (3.3%).

Calculations of count rates for each detected radionuclide depend on the establishment of secular equilibrium reached between ^238^U, and ^232^Th and for their decay products. Radioactivity concentrations of each sample were measured for about 48 h^16^.

### Radon measurement

**Figure 1:**
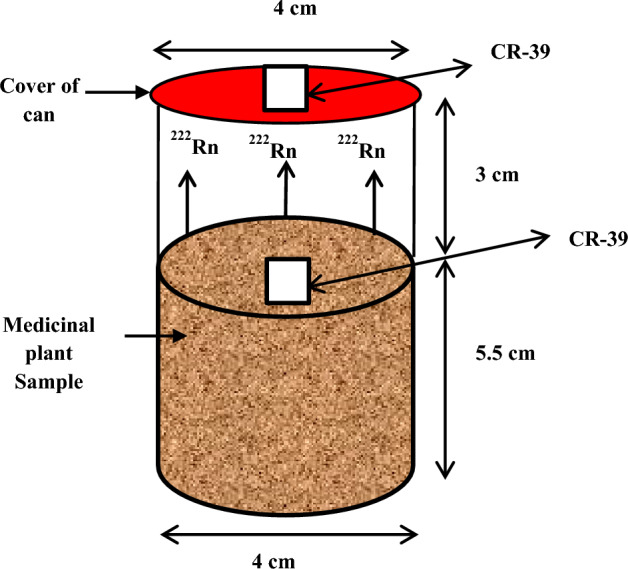
Sealed-cup technique for radon and thoron measurement.

Polyallyl Diglyol Carbonate (CR-39) nuclear track detector of 500 μm thickness with the chemical composition of (C_12_H_18_O_7_) and density of 1.31 g cm^−1^ was used in this work. A nuclear track detector has a high sensitivity to record the tracks of alpha particles, proton, and fission fragments because it has the bonds of weak carbon that break when exposed to ionizing radiation. The advantage of a nuclear track detector is the technology does not require sophisticated electrical equipment and is used for determining the radioactive substance due to their availability and accuracy^17^*.* A weighed amount of each sample was placed in plastic containers with dimensions 8.5 cm in height and 4 cm in average diameter. A piece of CR-39 detector of area 1 × 1 cm^2^ was embedded in the sample. At the same, time a second piece of the CR-39 detector was held at the top of the container (Fig. 1). The containers were left at room temperature for three months of exposure time. During this period alpha particles from the decay of radon, thoron, and their daughters bombard the CR-39 nuclear track detectors in the containers^18^. After, exposure the detectors were chemically etched in a 6.25N NaOH at 70 °C for 6 h to reveal the tracks, which were counted using an optical microscope. This experimental setup ensured that the detector in the bulk sample recorded alpha particles from radon, thoron, and their daughter products present in the investigated samples. The upper detector, however, only recorded the ^222^Rn component. Consequently, the difference in the track densities between the two detectors represents the content of ^220^Rn and daughters in the sample. The density of tracks counted was assumed proportional to the ^220,222^Rn exposure. The specific activity of radon in the investigated samples was calculated by using the following formula^19^

1 $$ C_{Rn} \left( {{\text{Bqm}}^{ - 3} } \right) = \frac{{\rho_{Rn} }}{\eta t} $$

where the variables: ρ_Rn_ is the radon track density (track cm^−2^), “η” is the efficiency factor for the CR-39 track detector this factor depended on the detector efficiency for the detection of alpha particles emitted from radon and its progeny^20^ and, “*t*” is the exposure time (90 days).

### Atomic absorption measurements

The levels of four toxic elements including Cupper (Cu), Zink (Zn), Cadmium (Cd), and lead (Pb) contained in six medicinal plant samples were estimated. In this study were analyzed using Atomic absorption spectrometry (AAS) which is widely used for the determination of trace elements in all kinds of samples. In Atomic absorption spectrometry, a beam of light passes the sample. Depending on the concentration of the element, a certain amount of light is absorbed. The concentration of the element can be estimated by comparing the intensity of the initial beam with the beam after passing the sample. The AAS instruments contain separate light sources for each element since each element absorbs light of a certain wavelength. AAS is often a technique for determining just one element per analysis.

### Statistical analysis

The results were statistically analyzed using the SPSS 10.0 software package (SPSS, Chicago, IL, USA) in accordance with accepted statistical practices. All measurements were performed in triplicates, where the average values (± standard deviation) were computed.

## Results

### Analysis of gamma spectrometry

**Figure 2:**
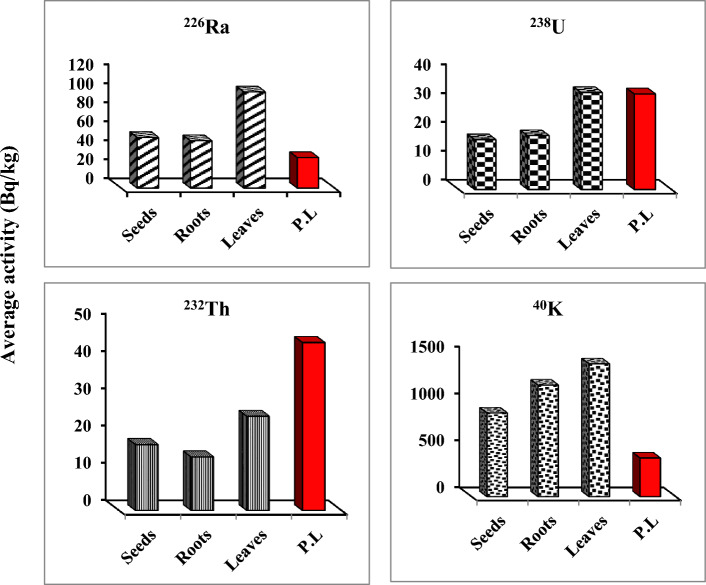
Average specific activity (Bqkg^−1^) of radionuclides in the medicinal plants.

**Figure 3:**
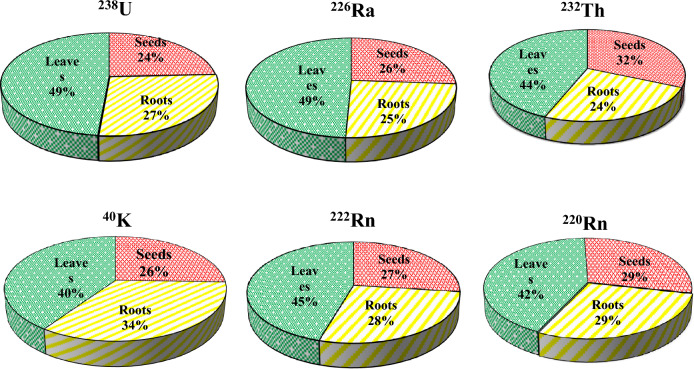
Contribution of radioactivity in some investigated medicinal plants.

Table 2 and Fig. 2 demonstrate the activity concentrations of natural radionuclides (^226^Ra, ^238^U, ^232^Th, ^40^K, ^222^Rn, and ^220^Rn) and their average values in the different medicinal plant samples. The highest values of these radionuclides (230 ± 19, 74 ± 2.5, 64 ± 6.6, 2440 ± 62, 4826 ± 96, and 2137 ± 43 Bqkg^−1^ respectively) were recorded in P9 (*Tilia europea*), which belong to leave the category. In, contrast the lowest value of ^226^Ra (29 ± 3.1 Bqkg^−1^) was recorded in P7 (*Pimpinella anisum*) which belong to the root category. Both ^238^U and ^40^K recorded their lowest value (9.7 ± 0.7 and 629 ± 13 Bqkg^−1^ respectively) in P3 and P14 which are belong to root and seed categories respectively. While ^232^Th was recorded as the lowest value (3.96 ± 0.9 Bqkg^−1^) in P7 (*Pimpinella anisum*) which belong to the root category. Also, the results showed that all radionuclides had high contribution in leaves samples than roots and seeds (Fig. 3).

**Table 2:** Specific activity (Bqkg^−1^) of radionuclides in medicinal plants.

Code	^238^U (Bqkg^−1^)	^226^Ra (Bqkg^−1^)	^232^Th (Bqkg^−1^)	^40^K (Bqkg^−1^)	^222^Rn (Bqm^−3^)	^220^Rn (Bqm^−3^)
P1	19.02 ± 1.0	49.04 ± 4.2	28.00 ± 1.5	400 ± 9.8	1522 ± 30	1230 ± 25
P2	16.33 ± 1.3	48.76 ± 4.7	11.09 ± 1.7	1137 ± 20	1425 ± 28	1166 ± 23
P3	09.72 ± 0.7	29.68 ± 2.1	08.04 ± 0.9	780 ± 13	1295 ± 25	1166 ± 23
P4	25.57 ± 1.6	59.40 ± 7.1	17.77 ± 2.2	1153 ± 27	2591 ± 51	1101 ± 22
P5	20.26 ± 1.7	58.96 ± 5.3	15.26 ± 1.6	1649 ± 26	2040 ± 40	1036 ± 21
P6	21.32 ± 1.5	66.95 ± 5.1	13.38 ± 1.8	794 ± 18	3239 ± 64	1004 ± 20
P7	07.50 ± 0.9	29.24 ± 3.1	3.96 ± 0.9	853 ± 17	939 ± 19	583 ± 12
P8	23.84 ± 1.7	80.42 ± 5.7	16.68 ± 2.0	1379 ± 25	3790 ± 75	1846 ± 37
P9	74.22 ± 5.4	230.31 ± 19	64.05 ± 6.6	2440 ± 62	4826 ± 96	2137 ± 43
P10	37.70 ± 2.3	89.80 ± 7.7	25.64 ± 2.7	1204 ± 27	4211 ± 84	1976 ± 39
P11	21.57 ± 1.6	74.18 ± 6.1	27.01 ± 2.1	951 ± 20	3628 ± 72	1716 ± 34
P12	18.15 ± 1.5	54.12 ± 4.8	11.58 ± 1.6	1753 ± 25	1976 ± 39	1036 ± 21
P13	17.39 ± 1.3	71.09 ± 5.2	11.58 ± 1.6	1422 ± 22	3304 ± 66	1522 ± 30
P14	14.63 ± 1.3	49.64 ± 3.6	11.58 ± 1.5	629 ± 13	1846 ± 37	1036 ± 21
P15	51.88 ± 4.2	160.62 ± 13	41.46 ± 4.4	1800 ± 42	4599 ± 92	1814 ± 36
P16	20.21 ± 1.6	58.44 ± 5.5	20.00 ± 1.8	872 ± 18	2526 ± 50	842 ± 17
P17	27.90 ± 1.8	65.29 ± 6.1	23.42 ± 2.1	1037 ± 21	3077 ± 62	1004 ± 20

### Assessment of radiological hazards

The radiological effects of radionuclides caused by internal and exterior alpha radiation can be evaluated using outdoor and indoor absorbed gamma dose rate (Dout and Din), outdoor and indoor annual effective doses (AEDE_out_ and AEDE_in_), radium equivalent activity (Raeq), internal hazard index (Hin), and outdoor and indoor excess lifetime cancer risk (ELCR_out_, ELCR_in_). In all calculations, A_Ra_, A_Th_ and, A_K_ are the activity concentrations of ^226^Ra, ^232^Th and, ^40^K in Bqkg^−1^.

### ***Radium equivalent activity (Ra***_***eq***_***)***

**Figure 4:**
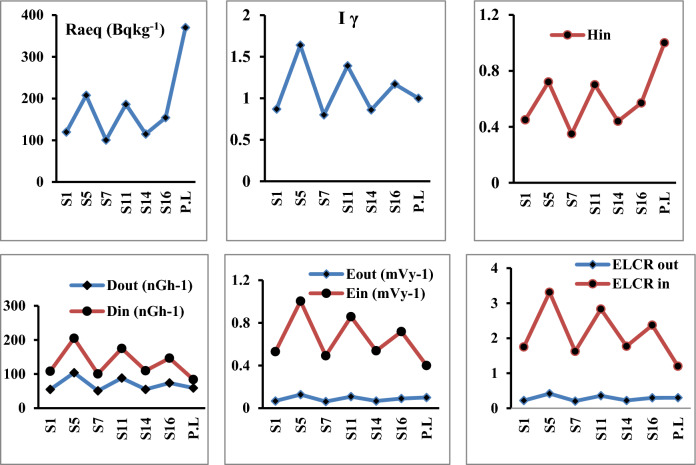
Radiological hazard indices in seeds samples.

**Figure 5:**
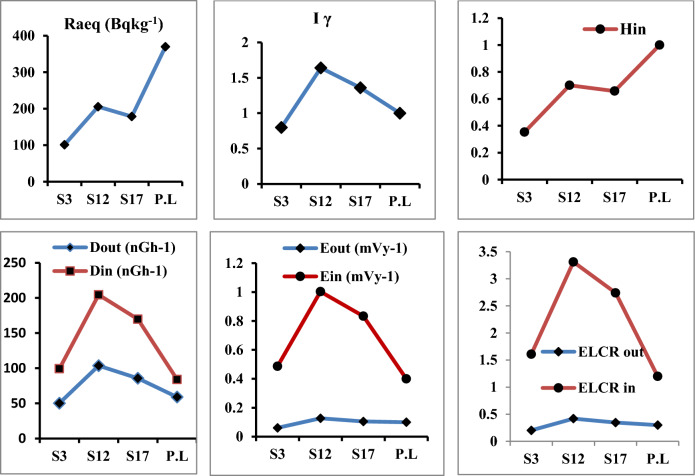
Radiological hazard indices in roots samples.

**Figure 6:**
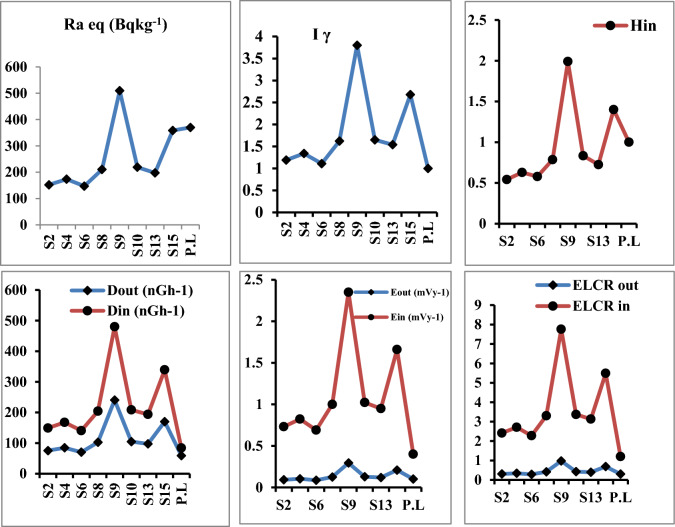
Radiological hazard indices in leaves samples.

The exposure to radiation has been defined in terms of radium equivalent activity (in Bqkg^−1^) and defined as the sum of the activity of ^238^U, ^232^Th, and ^40^K based on the assumption that 10 Bqkg^−1^ of ^238^U, 7 Bqkg^−1^ of ^232^Th, and 130 Bqkg^−1^ of ^40^K produced the same γ-ray dose rates and it used to compare the specific activity of materials containing different amounts of ^226^Ra, ^232^Th and, ^40^K in a single value^20^. The mean values of radium equivalent (Ra_eq_) in seed, root and leave samples were147.15 ± 2.7, 161.75 ± 3.0 and 245.93 ± 4.6 Bqkg^−1^ respectively Figs. 4, 5 and 6 which are lower than the recommended limit 370 Bqkg^−1^^21^.

### Assessment of outdoor and indoor absorbed gamma dose rate (D_out_ and D_in_)

The outdoor Dout and indoor Din absorbed gamma dose rates in nGyh^−1^ due to exposure to gamma radiation (emitted by ^238^U, ^232^Th, and ^40^K) were calculated in order to evaluate the radiological hazard of the natural radionuclides^10,22^ and recorded its highest values in leave samples (118.18 ± 2.3 and 235.34 ± 4.4 nGyh^−1^ respectively), which were higher than the worldwide average values (59 nGyh^−1^ for outdoor and 84 nGyh^−1^ for indoor) (Fig. 6)^10^*.*

### ***Outdoor and indoor annual effective dose rates (AEDE***_***out***_*** and AEDE***_***in***_***)***

The annual dose that individuals indoors and outdoors receive is known as an annual effective dose equivalent (AEDE). The calculated estimated dose provided by individuals inside and outside was calculated using a conversion factor of 0.7 Sv Gy1. People typically spend 80% of their time outside, whereas the remaining 20% of the time is spent inside their homes. Accordingly, the interior and outdoor occupancy variables were set to 0.8 and 0.2, respectively. AEDE_out_ and AEDE_in_ were estimated using the following equation^10,23^:

2 $$ {\text{AEDE}}_{{{\text{out}}}} \left( {\mu {\text{Svy}}^{{ - {1}}} } \right) = {\text{ D}}_{{{\text{out}}}} \times { 876}0\left( {{\text{hy}}^{{ - {1}}} } \right) \, \times \, 0.{2 } \times \, 0.{7 }\left( {{\text{SvGy}}^{{ - {1}}} } \right) $$

3 $$ {\text{AEDE}}_{{{\text{in}}}} \left( {\mu {\text{Svy}}^{{ - {1}}} } \right) = {\text{ D}}_{{{\text{in}}}} \times { 876}0\left( {{\text{hy}}^{{ - {1}}} } \right) \, \times \, 0.{8 } \times \, 0.{7 }\left( {{\text{SvGy}}^{{ - {1}}} } \right) $$

Also both AEDE_out_ and AEDE_in_ recorded their highest mean values (0.144 ± 0.002 and 1.153 ± 0.02 mSvy^−1^ respectively) in leave samples which were higher than the worldwide average values (0.07 mSvy^−1^ for outdoor and 0.410 mSvy^−1^ for indoor) (Fig. 6)^24^.

### Internal hazard index (H_in_)

In addition to exposure to radioactivity from the outside, radon and its short-lived byproducts are known to be harmful to human health, particularly the respiratory system^25^. The internal hazard index (H_in_)^26^ was used to measure the interior exposure to radon and its daughter products. For safe boundaries, the internal hazard index should be less than unity. In seed samples, roots, and leaves, the average readings of H_in_ were 0.538 ± 0.002, 0.570 ± 0.003, and 0.935 ± 0.01, respectively. As a result, the computed average values of the H_in_ index were below one.

### ***Gamma index (I***_***γ***_***)***

The European Commission^27^ has specified the gamma activity concentration index, I_**γ**_, as another measure of radiation hazard which defined as the risk arising from gamma radiation associated with radioactive natural nuclei in the investigated samples and calculated from equation depending on activity concentrations of ^226^Ra, ^232^Th and ^40^K. Its formula is as follows:

4 $$ \mathop I\nolimits_{\gamma } \,\, = \,\frac{{\mathop C\nolimits_{Ra} }}{150}\, + \,\frac{{\mathop C\nolimits_{Th} }}{100}\, + \,\frac{{\mathop C\nolimits_{K} }}{1500} $$

Because of the excessive external gamma radiation generated by natural radionuclides in the screened samples, the gamma index I_**γ**_ has a positive correlation with the yearly dose rate. In the current study, where the safety value for this index is 1, it was found that the maximum value was 1.86 ± 0.06 in leave samples.

### Outdoor and indoor excess lifetime cancer risk (ELCRout & ELCRin)

The following formulae were utilized to access the potential for outdoor and indoor cancer risk due to radiation exposure, while accounting for the average human age of 70 years^24,28^:

5 $$ {\text{ELCR}}_{{{\text{out}}}} = {\text{ E}}_{{{\text{out}}}} \times { 66 } \times 0.0{5} $$

6 $$ {\text{ELCR}}_{{{\text{in}}}} = {\text{ E}}_{{{\text{in}}}} \times { 66 } \times \, 0.0{5} $$

Also, the highest average values of outdoor and indoor excess lifetime cancer risk in leave samples were (0.47 ± 0.008 and 3.80 ± 0.07 respectively). Which are higher than the recommended levels of 0.29 × 10^–3^.

### Toxic element results

**Figure 7:**
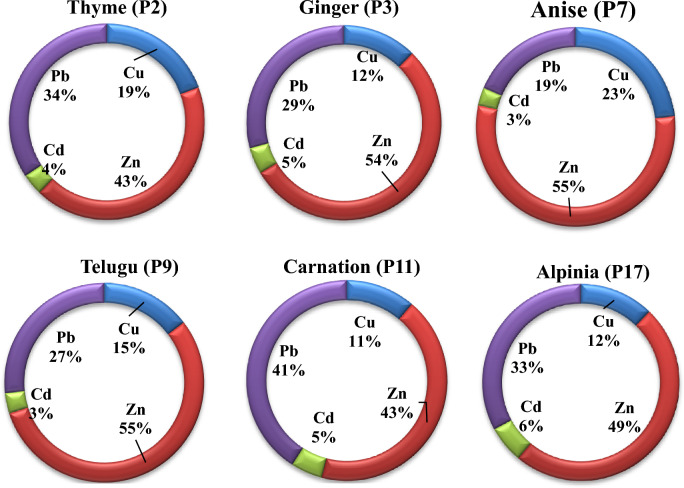
Contribution of toxic element in some investigated medicinal plants.

**Figure 8:**
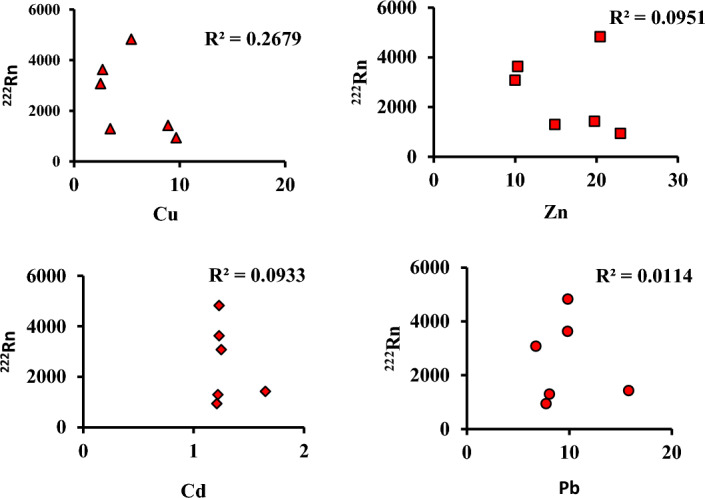
Relationship between toxic elements and ^222^Rn.

There are four toxic elements Cu, Zn, Cd and, Pb were determined in six medicinal plant samples (P2, P3, P7, P9, P11 and, P17) by flame atomic absorption spectrometry. Results show that Zink recorded the highest concentration in P7 (Ginger) 22.96 mg kg^−1^ and lead recorded the highest concentration in P2 (Thyme) at 15.80 mg kg^−1^. While the concentration of Cu ranged from 2.50 to 9.67 mg kg^−1^ and Cd from 1.21 to 1.65 mg kg^−1^ (Table 3). Both Zink and lead demonstrated the highest contribution among the investigated samples (Fig. 7). The relation between radon concentrations and four toxic elements was demonstrated in Fig. 8.

**Table 3:** Toxic elements in some medicinal plant samples.

Sample ID (name)	Cu mg kg^−1^	Zn mg kg^−1^	Cd mg kg^−1^	Pb mg kg^−1^
P2 (Thyme)	8.89	19.75	1.65	15.80
P3 Ginger	3.42	14.89	1.22	8.06
P7 Anise	9.67	22.96	1.21	7.73
P9 Telugu	5.42	20.45	1.23	9.86
P11 Carnation	2.70	10.32	1.23	9.83
P17 Alpinia	2.50	9.99	1.25	6.74

## Discussion

**Figure 9:**
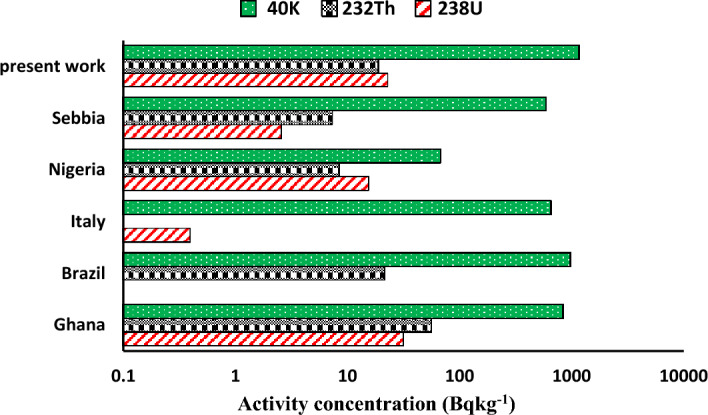
Comparison of mean specific activities of natural radionuclide in medicinal plants among different countries.

The average values of ^226^Ra in the examined medicinal plants are slightly higher than the recommended threshold of 33 Bqkg^−1^, but the average values of ^238^U in leaf samples are slightly higher than the typical world average value of 32 Bqkg^−1^. At the same, time ^222^Rn concentrations are much higher than the lower recommended level of ICRP (100–300 Bqm^−3^)^29^. While ^40^K is much higher than the reference value 412 Bqkg^−1^^30^. The high concentrations of ^40^K (892 ± 17,1190 ± 22, and 1416 ± 27 Bqkg^−1^) in seeds, roots, and leaves respectively may be due to unreasonable applications of potassium-containing fertilizer in soil from which medicinal plants absorb potassium in different amounts according to their metabolism. Also, the high concentrations of ^40^K are very significant because potassium is taken in through food and is completely absorbed upon ingestion, moving quickly from the gastrointestinal tract to the bloodstream. The variation in the activity concentrations of the different medicinal plants may be due to the fact that some of these plants tend to absorb more of certain elements than other plants. The activity concentration of a plant is also related to the soil, pH, and more generally the geographic region, in which the plant grows^31^. The highest concentrations of all radionuclides were found in P9 (*Tilia Europea*) from the leaves category, P11 (*Syzygium aromaticum*) from the seeds category and, P17 (*Alpinia officinarum*) from the roots category which is widely used for treating menstrual disorders as well as many other diseases. The mean activity concentration of ^238^U, ^232^Th and, ^40^K in the present work are compared with other works^11,32–35^ and are demonstrated in Fig. 9. This comparison show that the present study agrees with those measured in other reported countries, however the mean activity concentration of ^238^U in the present work are higher than those reported in Serbia^32^, Nigeria^33^, Italy^34^, and Brazil^35^ but is little less than those reported in Gana^11^. Also, ^232^Th in the present work is higher than reported countries except for Gana but ^40^K in the present work is significantly higher than reported countries.

From the present, study we found that some of the hazard indices are higher than the recommended level^36^ such as gamma index (I_γ_), outdoor and indoor excess lifetime cancer risk (ELCRout & ELCRin) however the workers in medicinal plants stores are externally exposed to radiation due to natural radionuclides present in these plants. Besides radioactivity toxic elements were determined in the investigated samples where both Zink and lead recorded high concentrations in the studied samples. The abnormal accumulation of these toxic elements in the human body is associated with health risks. Zink intoxication for example causes heart disease, night blindness, nausea, stomach pain, and diarrhea^37^. At the same time lead is known to cause bone marrow and kidney damage^38^. In, contrast Cu and Cd concentrations recorded lower levels in studied samples. A similar study^38^ determine trace elements (Cu, Fe, Zn, Mn, Cr, Ni, Pb, Se, As, and Cd) in Chinese herbal drugs. The authors found that the high concentration of Pb (0–6.59 μg g^−1^) and Zn (4.61–20.27 μg g^−1^) While Cupper (2.64–12.7 μg g^−1^) and Cadmium were not detected in the investigated samples. Also, similar study conducted in Turkey^39^ determined lead in 30 medicinal herbs. The author found that its concentration ranged from 0.67 to 2.59 μg g^−1^, in our, study this range was 6.74–15.80 μg g^−1^. This shows that the obtained values of lead were high than the range of this previous study. While both concentration ranges of Cadmium and lead 10 medicinal plants used in Pakistan^40^ were 0.59–1.66 and 3.15–10.63 μg g^−1^ respectively. In our, study the ranges of Cadmium and lead were 1.21–1.65 and 6.74–15.80 μg g^−1^ respectively. Our range for Pb is higher than those^40^. Poor negative correlations exist between ^222^Rn and four toxic elements in all investigated samples. These poor correlations may be due to the different geological behavior of radon and toxic elements. Notably, medicinal plants we investigated are controversially utilized in non-conventional medicine practicing. The range of exposure and the associated risk are determined by the rate of their consumption. Recently, after COVID-19 pandemic the combustion rate was increased worldwide, especially some literature evidenced the significant beneficial role of these plants and natural products (like phytocompounds) due to their substantial antiviral activity against SARS-CoV-2 and other coronaviruses^41^.

## Conclusions

Some medicinal plants daily used in Egypt were studied and the activity concentration of the naturally occurring radionuclides in medicinal plant samples was determined using hyper-pure germanium detectors and solid-state nuclear track detectors. The activity concentrations of naturally occurring radionuclides are higher than the average worldwide ranges in all leave samples. ^40^K recorded significantly higher concentrations in all samples. Also, all radiological parameters are higher in leave samples than the recommended allowable limit. Zn and Pb have the highest concentrations in all six samples under investigation. So Percussions must be taken into consideration for workers in these plant stores. Additionally, the data presented in this study will be used as a baseline to gauge how much radiation and toxic elements residents have been exposed to. The study revealed that there are no relations were found between radon concentrations and toxic elements in some investigated samples.

## Data Availability

Data sharing is not applicable to this article where the manuscript includes all the data we generated.
